# Oleuropein and punicic acid induce oxidative stress and cytotoxicity in MCF-7 breast cancer cells, with protection by peroxiredoxin antioxidant proteins

**DOI:** 10.1007/s11033-026-12952-y

**Published:** 2026-11-01

**Authors:** Samantha Diiorio, Braden Quitmeyer, Shelley A. Phelan

**Affiliations:** 1 Department of Biology, Fairfield University, Fairfield, CT USA; 2 Graduate Biomedical Sciences, University of Alabama, Birmingham, AL USA

**Keywords:** Breast cancer, MCF-7, Oleuropein, Punicic acid, Peroxiredoxin

## Abstract

**Background:**

Breast cancer remains a major cause of cancer-related mortality worldwide, creating a need for novel therapeutic strategies. Natural plant compounds have gained recent attention for their anticancer effects, including oleuropein from olive leaves, and punicic acid from the pomegranate fruit. In the present study, we investigated the effects of oleuropein (OL) and punicic acid (PA) on MCF-7 breast cancer cells and the role of peroxiredoxin (Prdx) antioxidants in mediating these effects.

**Methods:**

MCF-7 cells were treated with different concentrations of OL or PA for 72 h. Viable cell density was measured by MTT assay, cytotoxicity was measured by LDH release, mitochondrial ROS was measured by MitoSOX assay, and lipid peroxidation was measured by MDA assay. The effect of Prdx suppression was determined by transient siRNA transfection, followed by cell treatments and MTT assay.

**Results:**

Treatment with OL or PA produced a concentration-dependent reduction in cell viability, increased cytotoxicity, and morphological changes consistent with cellular stress and death, with PA exhibiting greater potency at lower concentrations. Both compounds significantly increased mitochondrial superoxide production, while PA also induced lipid peroxidation. Suppression of Prdx proteins increased cellular susceptibility to OL and PA treatment, with more pronounced effects following PA exposure.

**Conclusions:**

OL and PA induce oxidative stress and cytotoxicity in MCF-7 cells, and peroxiredoxins play a protective role in the cellular response. This study highlights possible efficacy of these agents for treatment, and the importance of antioxidant defense systems in subsequent cancer cell survival.

**Supplementary Information:**

The online version contains supplementary material available at https://doi.org/10.1007/s11033-026-12952-y.

## Introduction

Breast cancer is one of the most frequently diagnosed malignancies among women worldwide and is a leading cause of cancer-related mortality. In 2026, approximately 324,580 people will be diagnosed with breast cancer, and an estimated 42,670 deaths are expected to be reported [1]. Despite significant advances in diagnosis and treatment, resistance to conventional therapies and disease recurrence continue to present major clinical challenges. As a result, there is growing interest in identifying naturally occurring compounds that selectively target cancer cells while reducing toxicity to normal tissues. Natural products derived from dietary sources have emerged as promising candidates due to their diverse biological activities, including antioxidant, anti-inflammatory, and anticancer properties [2].

Oleuropein (OL) is the primary phenolic compound found in olive leaves and is considered a major contributor to the health benefits associated with the Mediterranean diet. Numerous studies have demonstrated that oleuropein exhibits anti-inflammatory, antimicrobial, antioxidant, and anticancer activities at dietary concentrations [3, 4]. Although traditionally regarded as an antioxidant, increasing evidence suggests that at higher concentrations, oleuropein can also exhibit pro-oxidant activity within cancer cells, promoting oxidative stress and cell death [5, 6]. These elevated concentrations have been shown to inhibit cell proliferation, induce apoptosis, and suppress tumor growth across a variety of cancer types including breast cancer cells [5–9]. For example, oleuropein significantly suppresses cell proliferation and induces apoptosis in the estrogen receptor-negative MDA-MB-231 and MDA-MB-468 lines [10, 11], and anti-metastatic effects have also been reported in the T-47D cell line, where oleuropein reduced migration and invasion through the induction of autophagy [12]. Furthermore, treatment with olive leaf extract, highly enriched in oleuropein, induced mitochondrial dysfunction, oxidative stress, and caspase-dependent apoptosis in MDA-MB-231 cells, supporting the role of oleuropein-mediated redox imbalance in cancer cell death [13]. Oleuropein-induced cytotoxicity and growth inhibition has also been observed in the ER+ MCF-7 cell line [10, 14, 15], and this susceptibility was further validated in mice with MCF-7 tumor xenografts [16]. Collectively, these findings suggest that oleuropein elicits anticancer effects through disruption of redox homeostasis, potentially overriding endogenous antioxidant defense systems.

Punicic acid (PA) is a conjugated omega-5 polyunsaturated fatty acid (18:3 n-5) and the predominant bioactive lipid found in pomegranate seed oil, accounting for approximately 65–80% of its fatty acid composition [17]. Increasing evidence has demonstrated that PA has potent anti-inflammatory, antioxidant, and anticancer activities [17, 18], although the molecular mechanisms responsible for its antitumor effects remain incompletely understood. Numerous studies have reported that PA inhibits proliferation and promotes apoptosis in a variety of cancer types through mechanisms involving oxidative stress, mitochondrial dysfunction, cell-cycle arrest, and modulation of multiple signaling pathways [17–19]. While very few studies have examined its effect on human breast cancer lines, Grossmann et al. (2010) demonstrated that PA inhibited the growth of both estrogen receptor-positive and estrogen receptor-negative breast cancer cells by more than 90% while inducing apoptosis through disruption of mitochondrial membrane potential [20]. The authors further showed that the antioxidant tocotrienol significantly reduced PA-induced cytotoxicity, suggesting that reactive oxygen species (ROS) generation and lipid peroxidation are important mediators of its anticancer activity [20]. These findings support PA as a promising anticancer compound whose therapeutic efficacy appears to depend, at least in part, on the balance between ROS generation and the antioxidant defense systems that enable cancer cell survival.

As noted above, both oleuropein and punicic acid act as antioxidants at low concentrations, but appear to “switch” to pro-oxidants at the higher concentrations used in these cellular studies. At physiological levels, reactive oxygen species (ROS) function as important signaling molecules for cell proliferation, differentiation, and survival, although excessive ROS accumulation can overwhelm cellular antioxidant defenses, resulting in oxidative stress and damage to proteins, lipids, and nucleic acids [21]. Many cancer cells maintain elevated basal ROS levels due to increased metabolic activity and rapid cell division, making them particularly vulnerable to additional oxidative stress [22]. Consequently, anticancer therapies can target this vulnerability by inducing ROS accumulation beyond a threshold compatible with cellular survival [22]. To counteract oxidative stress, cells possess several antioxidant defense systems. The peroxiredoxin (Prdx) family of enzymes are thiol-dependent antioxidant proteins that detoxify hydrogen peroxide, lipid hydroperoxides, and other reactive oxygen species while maintaining cellular redox homeostasis [23]. Humans express six peroxiredoxins (Prdx1–Prdx6), each with a particular cellular localization and tissue expression, contributing to distinct aspects of antioxidant defense. In addition to protecting cells from oxidative damage, peroxiredoxins regulate signaling pathways involved in cell growth, apoptosis, and tumor progression [23, 24]. Several studies have documented elevated levels of Prdx expression in cancers including breast cancer [23–26]. Furthermore, we previously reported Prdx overexpression in breast cancer tumor tissue [27] and demonstrated overexpression of five of the six Prdx genes in the MCF-7 cell line [28]. Together, these findings suggest that Prdx proteins likely help breast cancer cells survive under conditions of increased oxidative stress.

Our lab recently showed that treatment of MCF-7 cells with oleuropein significantly reduced cell viability and increased apoptosis while simultaneously increasing the expression of all six Prdx genes, suggesting that disruption of redox homeostasis may contribute to its anticancer effects in these cells [29]. Likewise, we also reported that punicic acid induces apoptosis in MCF-7 cells with a concomitant induction of Prdx expression [30, 31]. The adaptive upregulation of Prdx antioxidant enzymes may limit the effectiveness of OL or PA by counteracting excessive ROS accumulation, highlighting oxidative stress regulation as a critical determinant of cellular sensitivity to treatment. Thus, the purpose of the present study was to compare the effects of oleuropein and punicic acid in MCF-7 breast cancer cells and explore the role of peroxiredoxins in the cellular response. Specifically, we measured mitochondrial ROS production, lipid peroxidation, cell viability, and cytotoxicity in MCF-7 cells following treatment with different concentrations of OL and PA. In addition, siRNA mediated suppression of individual peroxiredoxin family members was used to evaluate the Prdx role in regulating cellular susceptibility to treatment. It was hypothesized that oleuropein and punicic acid would induce cytotoxicity in MCF-7 cells through increased oxidative stress and that suppression of peroxiredoxin expression would enhance cellular sensitivity to these compounds.

## Methods

### Cell culture

MCF-7 human breast adenocarcinoma cells were obtained from ATCC and cultured in Eagle’s Minimum Essential Medium supplemented with 10% fetal bovine serum (Corning) and 50 ng/mL insulin, maintained at 37 °C in a humidified atmosphere containing 5% CO₂. Cells were regularly split at 70–80% confluency and for all experiments cells were incubated under these conditions.

### Treatment conditions

Oleuropein was purchased from LKT Laboratories and reconstituted with 70% ethanol to make a 100 mg/ml stock solution. Punicic acid (9(Z),11(E),13(Z)-Octadecatrienoic Acid), was purchased from Cayman Chemical as a 10 mg/ml stock in methanol. Working solutions of oleuropein and punicic acid were prepared in 70% ethanol. The final treatment concentrations used for our experiments were 50 mg/mL (92.5 mM) oleuropein, 200 mg/mL (370 mM) oleuropein, 2 mg/mL (7.2 mM) punicic acid, 10 mg/mL (35.9 mM) punicic acid, and 50 mg/mL (179.5 mM) punicic acid. The final solvent concentration for all treatments was 0.35% ethanol, which was used as the control for all experiments. For all experiments, cells were seeded and incubated overnight at 37 °C in a humidified atmosphere containing 5% CO₂ for cell attachment, and treated the following day. All experiments were independently replicated at least three times.

### Cell morphology

Cells were seeded in 96-well plates at 2,000 cells per well and incubated overnight. The following day, cells were treated in replicates of 3 to 6 with 50 mg/mL oleuropein, 200 mg/mL oleuropein, 2 mg/mL punicic acid, 10 mg/mL punicic acid, 50 mg/mL or 0.35% etoh as vehicle control. Cells were incubated for 72 h. Cellular morphology was then evaluated using phase-contrast microscopy and images were acquired at 200X magnification using a Keyence digital imaging system. Morphological changes associated with cellular stress and cytotoxicity, including cell rounding, shrinkage, and reductions in cell density, were assessed qualitatively by comparing treated cells to ethanol treated controls.

### LDH cytotoxicity assay

Cytotoxicity was assessed using the CyQuant LDH Cytotoxicity Assay (Fisher Scientific). Cells were seeded in 96-well plates at 2,000 cells per well and incubated overnight. The following day, cells were treated in replicates of 3 to 6 with 50 mg/mL oleuropein, 200 mg/mL oleuropein, 2 mg/mL punicic acid, 10 mg/mL punicic acid, 50 mg/mL or 0.35% etoh as vehicle control and incubated for 72 h. At the time of assay, additional untreated wells designed for maximum LDH release were treated with lysis solution according to manufacturer’s protocol to lyse cells. Medias from all lysed, treated and control wells were then collected, incubated with LDH substrate mixture for 30 min at room temperature, and absorbance was measured at 490 nm (with background measured at 680 nm). Cytotoxicity was expressed as percentage relative to maximum LDH release controls.

### MTT cell viability assay

Viable cell density was measured using the MTT Cell Proliferation Assay (Cayman Chemical). Cells were seeded in 96-well plates at 2,000 cells per well and incubated overnight. The following day, cells were treated in replicates of 3 to 6 with 50 mg/mL oleuropein, 200 mg/mL oleuropein, 2 mg/mL punicic acid, 10 mg/mL punicic acid, 50 mg/mL or 0.35% etoh as vehicle control and incubated for 72 h. At the time of assay, the MTT reagent was added to each well and assayed according to the manufacturer’s protocol. Absorbance was measured at 570 nm, and viable cell density was calculated as a percentage of control wells.

### Mitochondrial superoxide detection (MitoSOX™ Red)

Mitochondrial superoxide levels were assessed using the MitoSOX™ Red mitochondrial Superoxide Indicator (InVitrogen). For all experiments, cells were seeded in 96-well plates at 10,000 cells per well, treated the following day in triplicate with 50 mg/mL oleuropein, 200 mg/mL oleuropein, 2 mg/mL punicic acid, 10 mg/mL punicic acid, 50 mg/mL or 0.35% etoh as vehicle control, and incubated for 24 h. At the time of assay, cells were incubated with the fluorescent MitoSOX Red working solution (prepared in HBSS) according to manufacturer’s protocol. Following incubation, cells were washed twice with warm HBSS and counterstained for 15 min with Hoechst, prepared as a 1:1,000 dilution in HBSS. Cells were subsequently imaged for red fluorescence, blue florescence and phase contrast optics using a Keyence digital microscope. The Keyence software was used to quantify total red fluorescence in each image.

### Lipid peroxidation assay (MDA)

Lipid peroxidation was quantified by measuring the Lipid Peroxidation malondialdehyde (MDA) Assay Kit (ApeXBio) according to manufacturer’s suggestions, with minor modifications. Briefly, cells were plated in 12-well plates and cells were grown to near confluency before treatment. Cells were treated in triplicate with 70% ethanol, 50 or 200 μg/mL oleuropein, or 2, 10, or 50 μg/mL punicic acid and incubated for 24 h. Cells were washed with PBS and lysed using the MPER mammalian protein extraction reagent (ThermoScientific) on ice to preserve sample integrity. Lysates were centrifuged, and supernatants were collected for analysis with the MDA assay. MDA standards were prepared in MPER across a defined concentration range and combined with a freshly prepared TBA working solution containing thiobarbituric acid (TBA), dilution buffer, and antioxidant. Both standards and samples were assayed according to manufacturer’s instructions. Absorbance of the final supernatants were measured at 532 nm. To ensure comparability across samples, MDA concentrations were normalized to total protein content, which was determined in parallel using a colorimetric Coomassie-based Bradford Protein Assay (Pierce) assay, with absorbance measured at 595 nm.

### Peroxiredoxin suppression

Targeted knockdown of peroxiredoxin (Prdx) genes (Prdx1–Prdx6) was performed using transient siRNA transfection. Cells were seeded in 96-well plates at 10,000 cells per well and triplicate wells assigned for each condition. Twenty four hours after seeding, cells were transfected in Opti-MEM using Lipofectamine 2000 in combination with Prdx-specific siRNA constructs (Prdx1–Prdx6) or a non-targeting negative control siRNA. For each condition, siRNA-lipid complexes were prepared by separately diluting 10 pmol of siRNA and 0.5ul Lipofectamine 2000 in a total of 50 ul of Optimum medium, followed by a 20-min incubation prior to addition to cells. After transfection, cells were then incubated for 48 h and subsequently treated with 50 mg/mL of oleuropein or 10 mg/mL of punicic acid and incubated for an additional 72 h at 37 °C in 5% CO₂. After treatment, cells were assayed for viable cell density using an MTT assay.

### Measurement of peroxiredoxin expression

To measure peroxiredoxin expression, transfected cells were lysed 48 h after transfection, and RNA was isolated and reverse transcribed using the Cells-to-CT kit (Applied Biosystems) according to manufacturer’s protocol. Taqman Real Time PCR Assays were conducted using Applied Biosystems primer/probes specific to each of the six mammalian peroxiredoxin genes. These included human-specific TaqMan assays for Prdx1 (Hs00602020), Prdx2 (Hs03044902), Prdx3 (Hs00428953), Prdx4 (Hs00197384), Prdx5 (Hs00738905) and Prdx6 (Hs00705355). An 18S rRNA primer/probe (Applied Biosystems cat#4333760F) was used as an endogenous control for all PCR reactions. All reactions were set up in triplicate and were used to calculate relative quantification (RQ).

### Statistical analysis

For each experiment, data were statistically analyzed by comparing the mean of each experimental group with that of the appropriate control group using individual Student’s T-tests. For each analyzed data set, statistical significance was determined with a p value <0.05.

## Results

### Concentration-effect of oleuropein and punicic acid on MCF-7 morphology

**Fig. 1:**
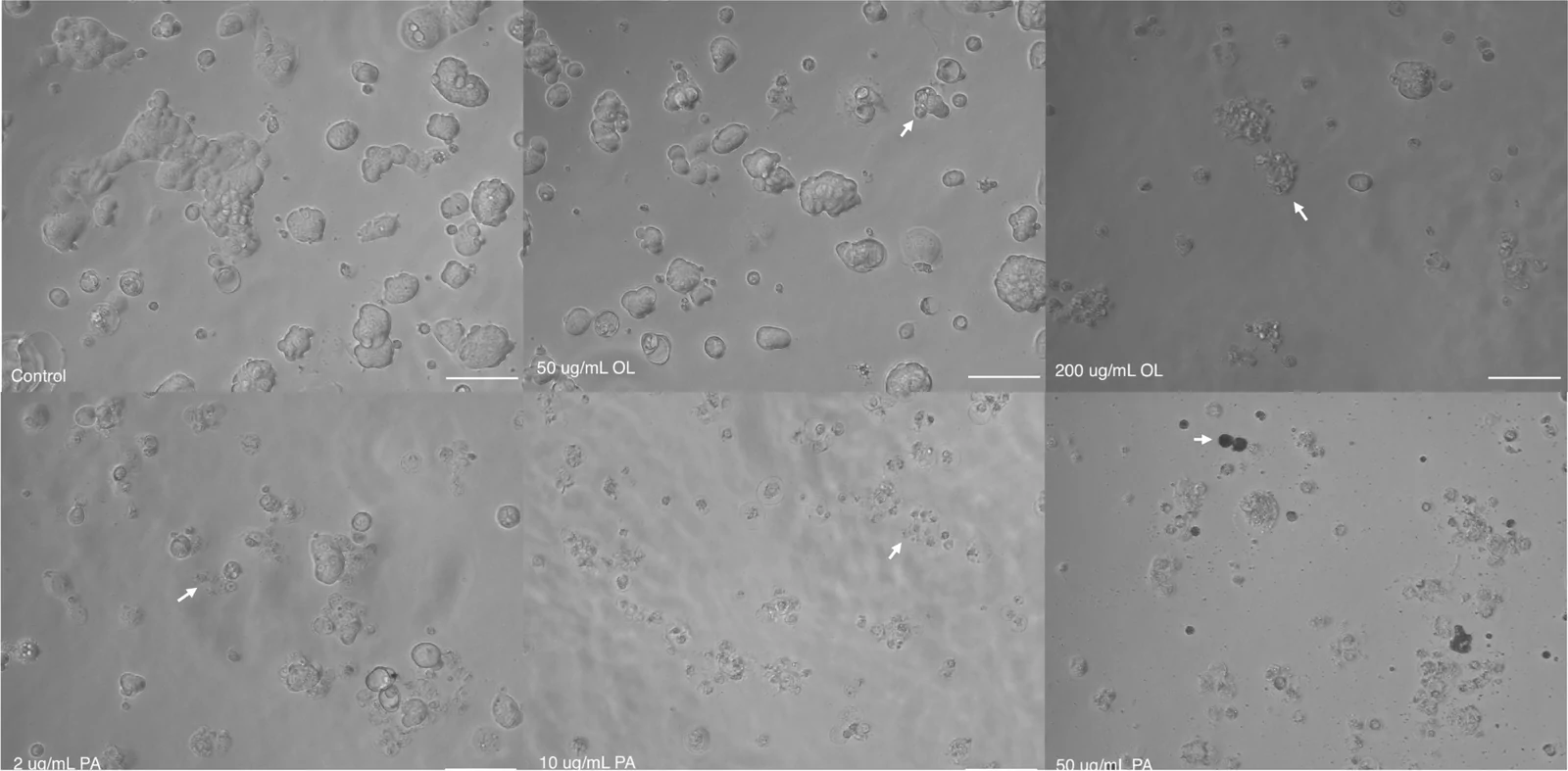
Effect of oleuropein (OL) an punicic acid (PA) on MCF-7 cellular morphology. Representative phase-contrast images of MCF-7 cells treated with ethanol (ETOH), 50 or 200 mg/mL Oleuropein (OL), or 2, 10, or 50 mg/mL punicic acid (PA) for 72 h. Images were acquired at 200× magnification using an inverted light microscope. Scale bars = 100*μ M*

To determine whether oleuropein (OL) and punicic acid (PA) induce morphological changes associated with cytotoxicity, MCF-7 cells were treated with increasing concentrations of each compound and visualized by phase-contrast microscopy after 72 h. We selected these particular concentrations and timepoint for consistency with our previously published work (29–31). As shown in Fig. 1, control cells displayed normal morphology with extensive cell–cell contact and the typical cobblestone appearance. We confirmed that the 0.35% ethanol vehicle control had no effect on MCF-7 cells (Fig. S1). In contrast, treatment with both OL and PA resulted in concentration-dependent morphological changes. Cells exposed to 50 mg/mL exhibited slightly more rounded appearance, while 200 mg/mL OL caused noticeable cell shrinkage and reduced confluency compared to controls. Similar concentration-dependent morphological changes were observed following treatment with punicic acid, but at lower concentrations. Subtle changes were observed at 2 mg/mL but substantial cell shrinkage was seen at 10 mg/mL PA. Much more severe effects were observed at 50 mg/mL PA, with extensive condensation and cell loss. These findings show that both compounds negatively impact MCF-7 cell viability, with PA having comparably greater cytotoxicity effects at lower concentrations.

### Concentration-effect of oleuropein and punicic acid on MCF-7 cell growth and viability

**Fig. 2:**
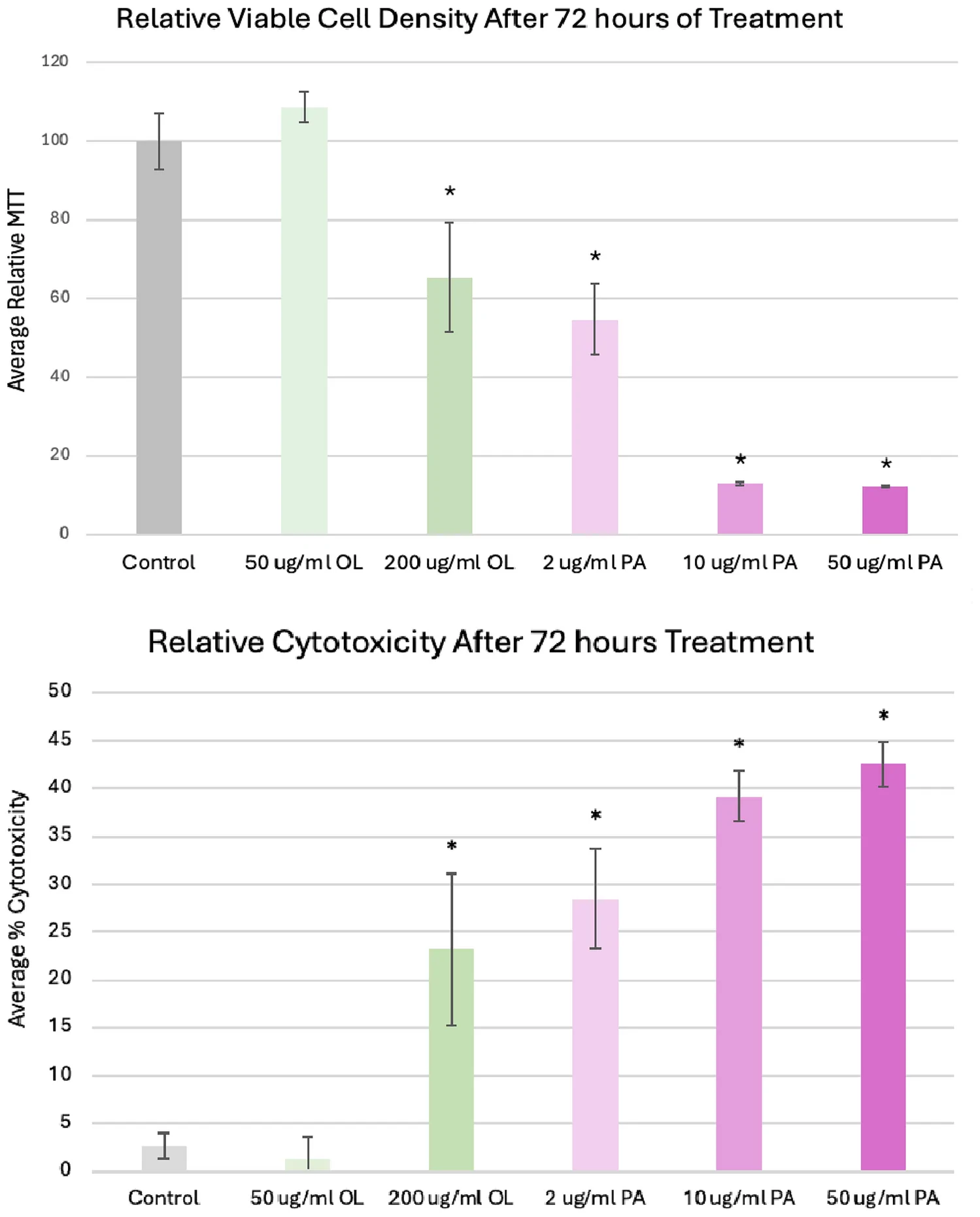
Effects of oleuropein and punicic acid on MCF-7 Cell density & viability. MCF-7 cells were treated with ethanol, 50 or 200 mg/mL oleuropein, or 2, 10, or 50 mg/mL punicic acid for 72 h. (Top) viable cell density was assessed using an MTT assay, with final absorbance measured at 570 nm, normalized to the ethanol control. (Bottom) Relative cytotoxicity was assessed using an LDH release assay. Absorbance was measured at 490 nm with background measured at 680 nm, and % cytotoxicity calculated. All data represent mean values from replicate samples (n = 3) ± standard deviation. *p < 0.05 compared with ethanol-treated controls

The effects of OL and PA on cell growth and viability were measured at 72 h in two different ways. First, an MTT assay was used to measure viable cell density following 72 h of treatment (Fig. 2). As shown, both compounds significantly reduced viable cell density relative to untreated controls in a concentration-dependent manner. For oleuropein, the 50 mg/mL concentration had no effect, yet 200 mg/mL oleuropein led to a nearly 40% reduction in viable cell density. In contrast, even the lowest concentration of punicic acid reduced viable cell density by nearly 50%, with almost 90% reduction in viable cell density with the two higher concentrations.

Since the MTT assay can be affected by both cytotoxicity and inhibition of proliferation, we wanted to directly measure cytotoxicity using an LDH release assay, which measures LDH released into the media as a result of loss of membrane integrity (Fig. 2). Control-treated cells showed the expected low background cytotoxicity (below 5%). Consistent with the MTT results, treatment with increasing concentrations of OL or PA resulted in increased LDH release relative to controls, indicating compromised membrane integrity and cellular death. Again, the 50 mg/mL oleuropein treatment had no measurable effect, while 200 mg/mL oleuropein led to a significant increase in cytotoxicity to approximately 23%. With punicic acid treatment, cytotoxicity increased in a concentration-dependent way, with 2 mg/mL producing approximately 28% cytotoxicity, 10 mg/mL producing 38% cytotoxicity, and 50 mg/mL producing 42% cytotoxicity. Together, the MTT and LDH findings demonstrate that OL and PA reduce MCF-7 cell survival.

### Induction of oxidative stress in MCF-7 cells by oleuropein and punicic acid

**Fig. 3:**
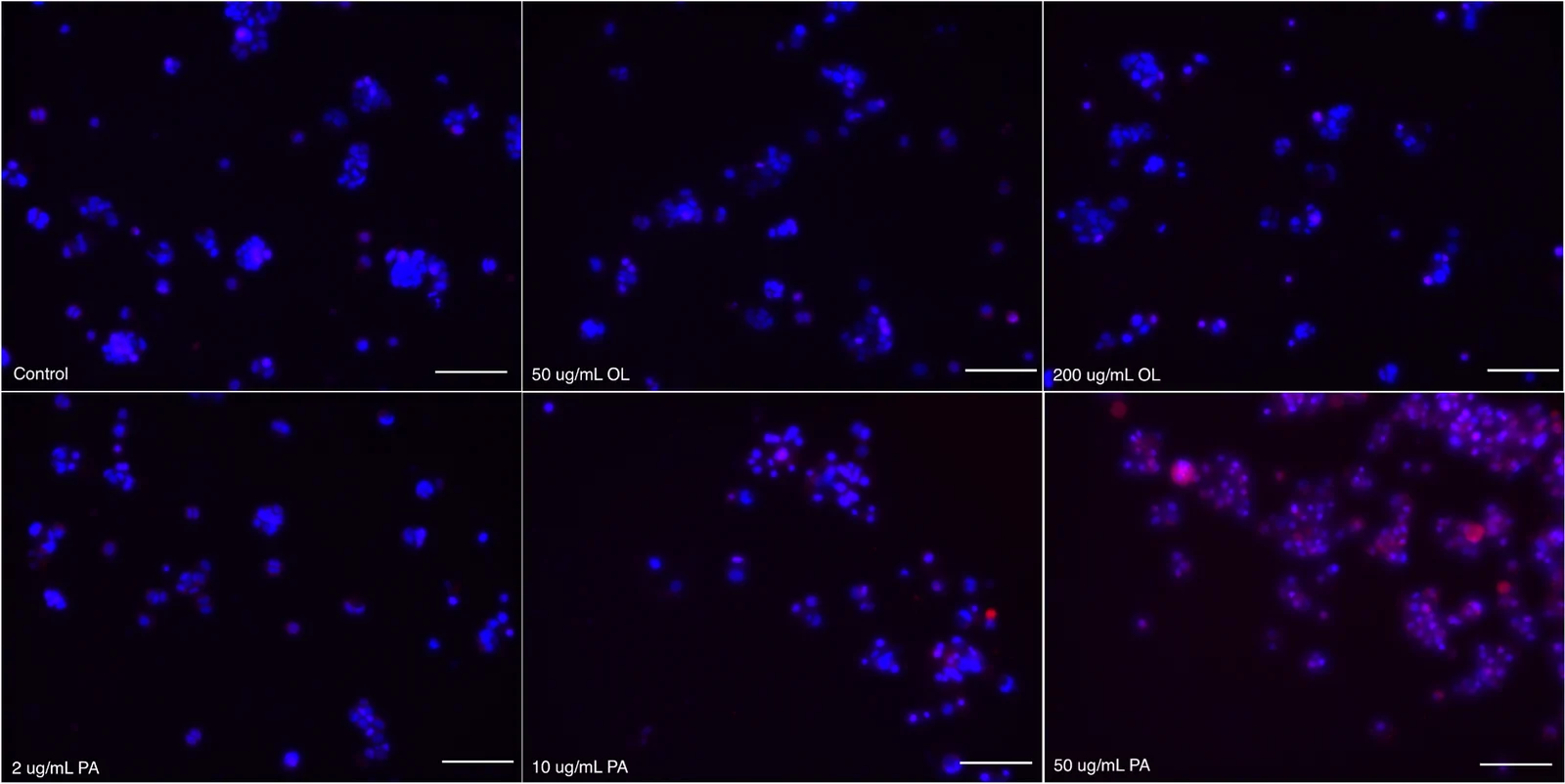
Effect of oleuropein or punicic acid on mitochondrial reactive oxygen species in MCF-7 cells. Representative fluorescence microscopy images of MCF-7 cells treated with ethanol (ETOH), 50 or 200 mg/mL oleuropein (OL), or 2, 10, or 50 mg/mL punicic acid (PA) for 24 h. Following treatment, cells were stained with MitoSOX Red to detect mitochondrial superoxide production (red) and Hoescht nuclear stain to visualize nuclei (blue). Images were acquired at 200X magnification using a fluorescence microscope under consistent exposure settings. Scale bars = 100*μ M*

To investigate whether oxidative stress contributes to the observed cytotoxicity, mitochondrial superoxide production was measured after 24 h of treatment using the MitoSOX assay, using Hoechst as a counterstain (Fig. 3). Control cells showed minimal detectable red fluorescence, indicating low mitochondrial superoxide production. In contrast, treatment with either OL or PA resulted in visibly increased MitoSOX fluorescence with increasing dosage. The lowest concentration of oleuropein had little increase in mitochondrial superoxide levels, with some increase at 200 mg/mL OL. With punicic acid, the lowest 2 mg/mL concentration had minimal mitochondrial superoxide fluorescence, but a noticeable increase at 10 mg/mL. At the 50 mg/mL PA concentration, mitochondrial superoxide levels were very high as is evident by the intense red/blue co-staining. Therefore, mitochondrial superoxide levels increased in a concentration-dependent manner for both compounds, with substantially larger effects with punicic acid. Phase contrast images corresponding to the fluorescent images are included in supplemental material (Fig. S2).

### Induction of lipid peroxidation in MCF-7 cells by punicic acid

**Fig. 4:**
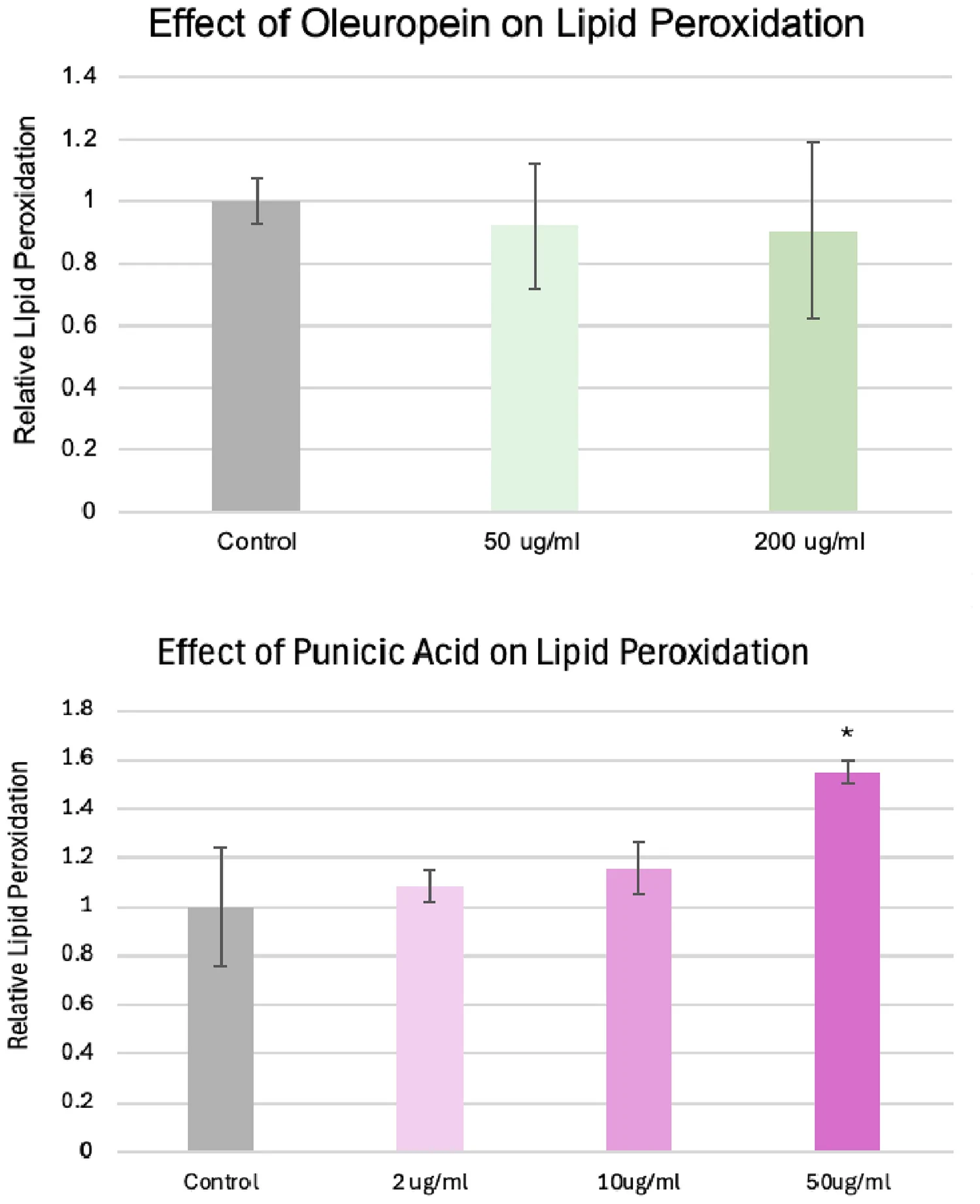
Effect of oleuropein and punicic acid on lipid peroxidation in MCF-7 cells relative lipid peroxidation in MCF-7 cells following treatment with 70% ethanol vehicle control, 50 or 200 mg/mL OL, or 2, 10, or 50 mg/mL PA for 24 h. Lipid peroxidation was quantified using a malondialdehyde (MDA) assay according to the manufacturer’s protocol. Absorbance was measured and normalized against protein concentration. All data represent mean values from replicate samples (n = 3) ± standard deviation. *p < 0.05 compared with vehicle treated controls. Results are displayed oleuropein (top) and punicic acid (bottom)

To determine whether either oleuropein and/or punicic acid may result in oxidative damage to cellular membranes, lipid peroxidation was assessed by measuring malondialdehyde (MDA) levels following 24 h of treatment. As shown in Fig. 4, OL treatment did not increase MDA levels relative to untreated controls. In contrast, while the lower concentrations of punicic acid showed no significant increase in MDA levels, the highest concentration of punicic acid induced a nearly 50% increase in lipid peroxidation after 24 h of treatment. PA treatment significantly increased MDA production compared to controls (Fig. 4).

### Establishment of peroxiredoxin suppression by transient siRNA transfection

**Fig. 5:**
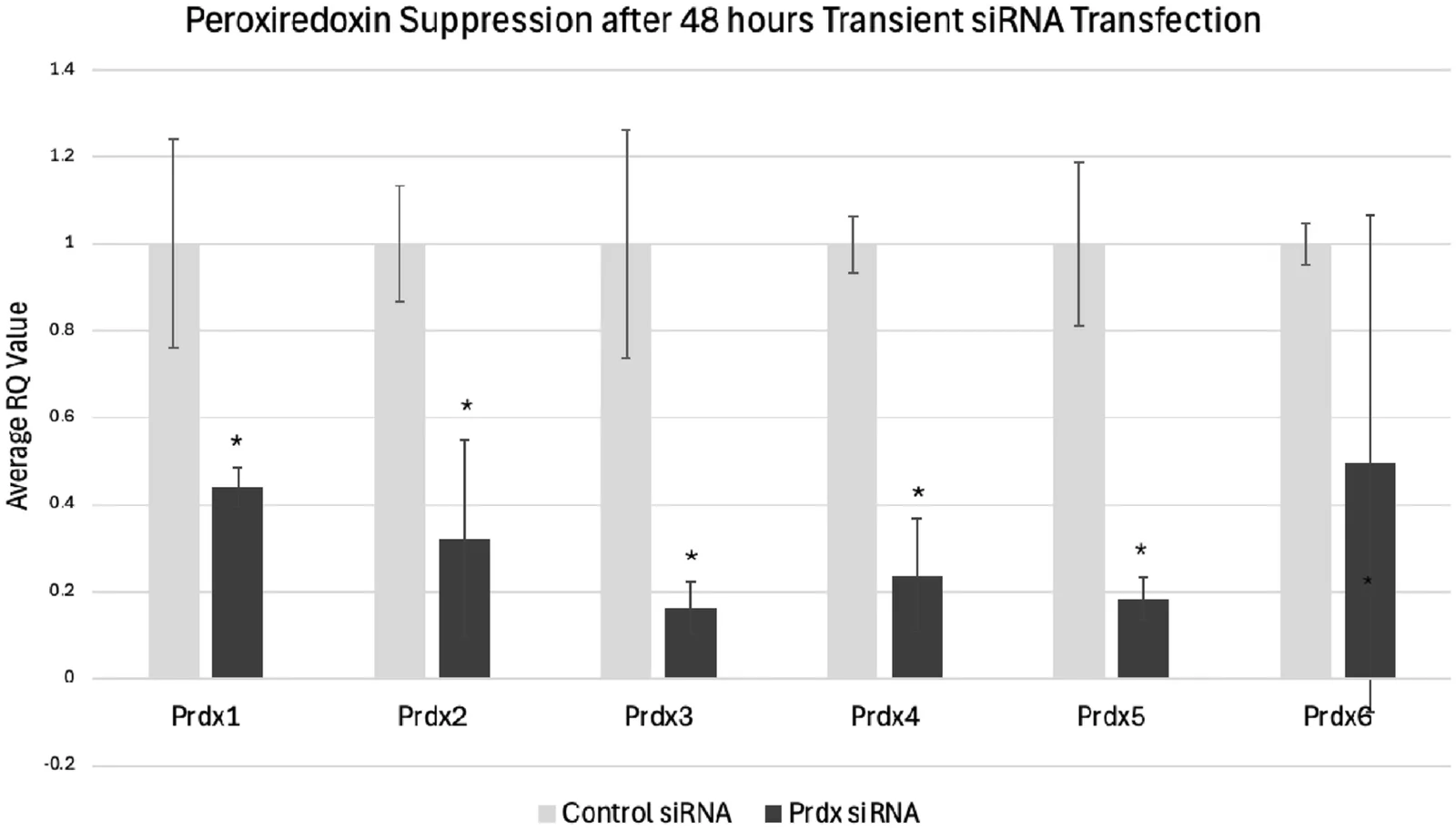
Prdx suppression by siRNA transfection. MCF-7 cells were transfected (in triplicate) with the indicated Prdx siRNA and mRNA expression for the target Prdx gene was assayed 48 h after transfection using qPCR in triplicate reactions. Average relative quantification (RQ) from replicate reactions (n=3) normalized to 18S levels is shown ± standard deviation for experiments representing maximal suppression. *p < 0.05 compared with negative controls siRNA-tranfected cells for each gene

We previously reported induction of the Prdx gene family in response to both oleuropein and punicic acid [29–31]. To begin to explore the role of each Prdx protein in the cellular response to these chemicals, we sought to suppress the expression of each Prdx protein using siRNA-mediated knockdown. MCF-7 cells were transfected with siRNA targeting individual peroxiredoxin (Prdx) mRNAs and expression levels were quantified by qPCR 48 h after transfection. Expression levels were normalized to cells transfected with a negative control siRNA. As shown in Fig. 5, transient transfection with Prdx-specific siRNA significantly reduced expression of Prdx1–Prdx5 compared to the respective negative control. Prdx1 expression was reduced to approximately 43% of control levels, while Prdx2 expression was reduced to approximately 32%. The greatest suppression was found for Prdx3, Prdx4 and Prdx5, with expression reduced to approximately 15%, 23% and 17%, respectively. Transient transfection targeting Prdx6 resulted in a highly variable suppression in all experiments, with no significant reproducible reduction in expression. These results validated our transfection protocol for Prdx1-5, providing an experimental model for examining their role in cellular resistance to OL and PA.

### Effect of peroxiredoxin suppression on MCF-7 susceptibility to oleuropein and punicic acid

**Fig. 6:**
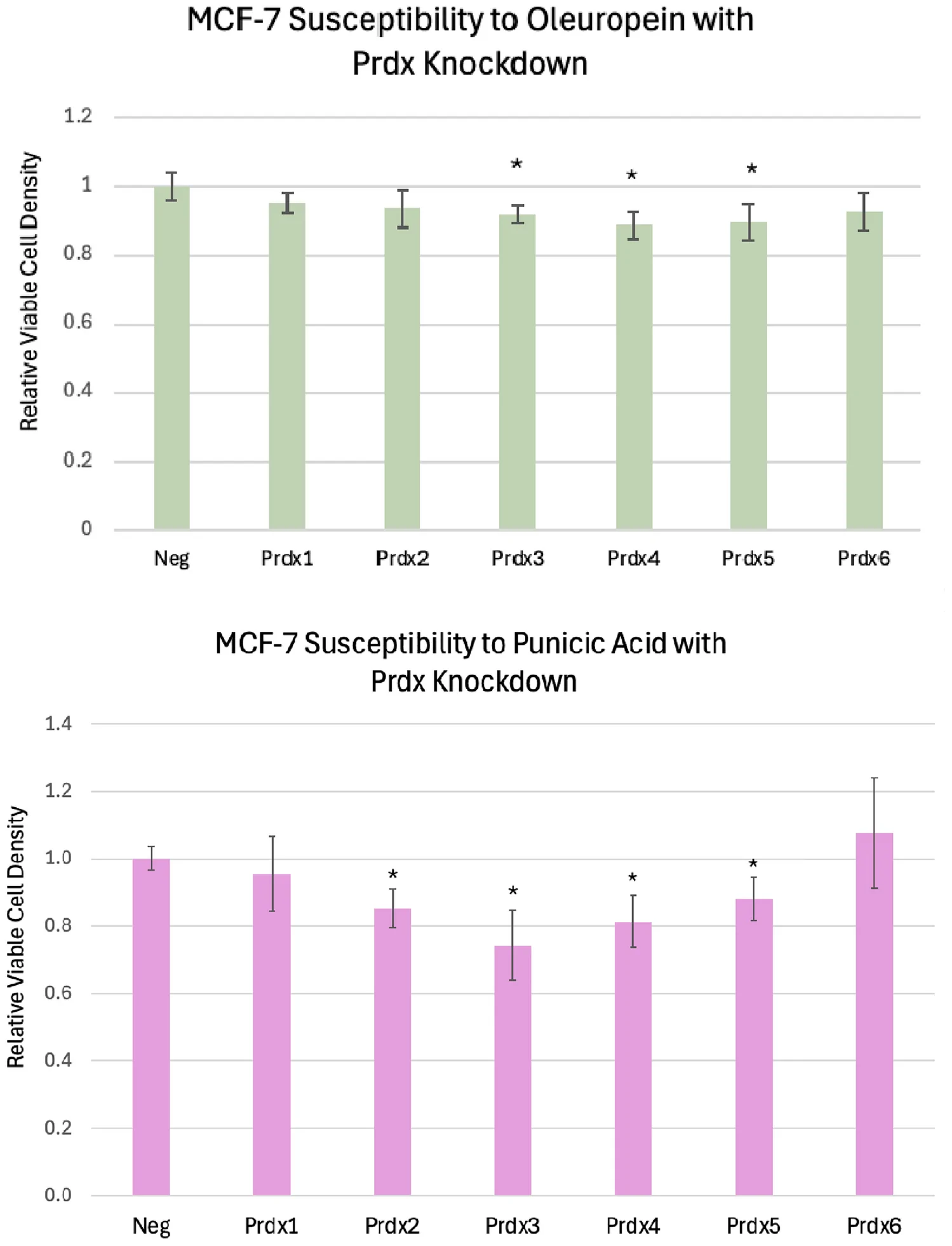
Effect of Prdx suppression on MCF-7 susceptibility to oleuropein and punicic acid. MCF-7 cells were transfected with negative control siRNA or siRNA targeting individual Prdxs (Prdx1–Prdx6). 48 h after transfection, cells were treated with either 50 mg/mL Oleuropein (top) or 10 mg/mL punicic acid (bottom) for 72 h. Cell viability was assessed using an MTT assay, and results are expressed as relative viable cell density normalized to the negative control siRNA group. All data represent mean values from replicate samples (n=3) ± standard deviation. *p < 0.05 compared to the negative control siRNA group

To determine whether suppression of individual Prdx family members altered cellular responses to OL or PA, MCF-7 cells transiently transfected with Prdx-specific siRNAs were subsequently treated with each compound for 72 h and viable cell density measured by MTT assay following treatment. We chose 50 mg/ml OL and 10 mg/ml PA since these were below the highly cytotoxic concentrations for the two chemicals, allowing us to see any further reduction in viability as a result of Prdx suppression. As shown in Fig. 6, suppression of Prdx1, Prdx2 and Prdx6 produced no effect on oleuropein susceptibility, while suppression of Prdx3, Prdx4, and Prdx5 resulted in small but significant reductions in viable cell density in response to oleuropein, with relative viable cell densities of 0.92, 0.89 and 0.9, respectively. Following PA treatment, significant decreases in viable cell density were observed following suppression of Prdx2, Prdx3, Prdx4, and Prdx5, with relative viable cell density in response to PA falling to approximately 0.5, 0.74, 0.81 and 0.88, respectively. Overall, suppression of Prdx2, Prdx3, Prdx4 and Prdx5 significantly increased susceptibility to these compounds. Representative phase contrast images of wells used in these experiments is included in supplemental material (Fig. S3).

## Discussion

This study investigated whether oleuropein and punicic acid induce cytotoxic effects in MCF-7 breast cancer cells through similar mechanisms of oxidative stress and whether the peroxiredoxin antioxidant defense system may play a protective role against these effects. The results support the primary hypothesis by demonstrating that these compounds reduce cell viability, increase cytotoxicity, and elevate mitochondrial ROS production, but show that only PA induces lipid peroxidation. Additionally, our data show a functional role of peroxiredoxins in MCF-7 cell protection to these chemicals.

Morphological assessment demonstrated that both oleuropein (OL) and punicic acid (PA) induced pronounced cellular stress in MCF-7 cells, characterized by reduced confluency, cellular shrinkage, and loss of normal morphology, with these effects becoming increasingly evident at higher treatment concentrations. These observations are consistent with previous reports demonstrating that olive-derived polyphenols promote apoptotic morphological changes in breast cancer cells through activation of intrinsic cell death pathways and disruption of mitochondrial function [32]. While reductions in cell viability measured by the MTT assay were accompanied by increased LDH release, the magnitude of these responses differed. This suggests that these compounds may not only cause cytotoxicity but may also inhibit proliferation. This is particularly true following PA treatment. For example, the highest concentrations of PA reduced viable cell density by nearly 90%, whereas LDH release increased by only 35–45%. This discrepancy suggests that PA may also inhibit cellular proliferation in addition to cytotoxicity, an interpretation supported by previous studies demonstrating that punicic acid suppresses breast cancer cell growth through cell-cycle arrest and activation of apoptotic signaling rather than exclusively causing acute membrane lysis [20]. Interestingly, PA produced visible morphological damage at lower concentrations than OL, suggesting greater cytotoxic potency under the conditions examined. This observation is consistent with other reports describing conjugated linolenic acids, including punicic acid in the 10–100 uM range, as potent inducers of oxidative stress, mitochondrial dysfunction, and apoptosis in prostate cancer cells, resulting in rapid suppression of tumor cell growth [33]. Collectively, these findings support the conclusion that both compounds exert significant antitumor activity in MCF-7 cells through overlapping but potentially distinct mechanisms and suggest that punicic acid may be a better treatment candidate given its higher efficacy at lower concentrations.

A major objective of this study was to compare the role of oxidative stress in the anticancer activity of OL and PA. Our results demonstrated a concentration-dependent increase in mitochondrial superoxide following treatment with either compound, with significantly greater potency observed following PA treatment. Because mitochondria are a major source of intracellular ROS, excessive mitochondrial ROS production can disrupt cellular homeostasis, damage biomolecules, and activate cell death pathways [34]. The magnitude of the elevated mitochondrial ROS levels at 24 h preceded the cytotoxicity observed at 72 h, suggesting that oxidative stress represents an early cellular response to treatment rather than a consequence of cell death. Similar relationships have been reported for numerous anticancer agents, in which ROS generation occurs before measurable apoptosis and serves as an initiating signal for mitochondrial dysfunction and cell death [35, 36]. Although oleuropein is widely recognized for its antioxidant properties in normal tissues, accumulating evidence indicates that certain concentrations can exert pro-oxidant effects in cancer cells by increasing intracellular ROS, disrupting mitochondrial homeostasis, and activating apoptotic signaling pathways [32, 37]. Likewise, growing evidence suggests that the anticancer activity of punicic acid is closely associated with modulation of oxidative stress, mitochondrial dysfunction, and apoptosis in multiple cancer models, supporting a role for ROS-mediated mechanisms in its biological activity [38]. While we were unable to quantify mitochondrial superoxide levels with this assay, our microscopy did show increased fluorescence with 10 and 50 mg/ml punicic acid, which is consistent with the subsequent increase in cytotoxicity observed by 72 h. Consistent with published reports, punicic acid produced substantially greater mitochondrial superoxide accumulation than oleuropein in the present study, suggesting that enhanced oxidative stress may contribute to its stronger anticancer activity in MCF-7 cells.

Further support for this mechanism was provided by our lipid peroxidation data. Increased MDA levels following the high concentration treatment with punicic acid indicate that elevated ROS production results in oxidative damage to cellular lipids at this concentration. Lipid peroxidation can compromise membrane integrity, alter membrane-associated signaling pathways, and generate secondary reactive aldehydes that further amplify cellular damage [39]. The observed effects of punicic acid on lipid peroxidation are consistent with the increased mitochondrial ROS and cytotoxicity observed at this concentration. This ability of PA to elevate both mitochondrial ROS production and lipid oxidation supports a mechanistic link between oxidative stress and the cytotoxic effects observed in MCF-7 cells. Since the 10 mg/ml concentration did not show lipid peroxidation, but did show increased mitochondrial superoxide and cytotoxicity, it is possible that this concentration was not sufficient to oxidize lipids within 24 h. Recent evidence shows that the highly conjugated structure of punicic acid renders it particularly susceptible to oxidation, promoting the accumulation of oxidized lipids that can amplify oxidative stress, disrupt mitochondrial function, and activate ferroptosis in prostate cancer cells [33] and carcinoma cells [40]. This amplification of lipid peroxidation provides a plausible explanation for the mechanism of action by which punicic acid exerts its effects on MCF-7 cells.

To further investigate the role of antioxidant defense systems in protecting MCF-7 cells from oxidative stress, individual Prdx family members were suppressed using transient siRNA transfection. Quantitative PCR analysis confirmed successful knockdown of Prdx1 through Prdx5. Overall, knockdown of specific Prdxs increased cellular susceptibility to both compounds, although the magnitude of the effect differed between treatments and Prdx targets. Suppression of Prdx3, Prdx4, and Prdx5 slightly but significantly reduced viable cell density in response to oleuropein. While these reductions in viability were relatively modest, the results do suggest that these proteins contribute to protection against oleuropein induced cellular stress. More substantial effects were observed following PA treatment, with suppression of Prdx2, Prdx3, Prdx4, and Prdx5 more substantially reducing cell viability, indicating increased sensitivity to PA induced cytotoxicity when antioxidant defenses were impaired. While transfection with Prdx1 and Prdx6 showed no significant effect on viable cell density in response to either compound, the level of suppression achieved for these two genes was substantially less and varied more than the others, potentially explaining no measurable effect. As a result, we cannot conclude whether Prdx1 or Prdx6 play a protective role against oleuropein or punicic acid.

The enhanced susceptibility observed following Prdx2-5 suppression supports the proposed role of peroxiredoxins as protective antioxidant enzymes in MCF-7 cells. Peroxiredoxins constitute an important cellular defense system that detoxifies hydrogen peroxide and other reactive oxygen species, thereby maintaining redox homeostasis [41]. The increased sensitivity of Prdx-deficient cells to OL and PA is consistent with the earlier observations that both compounds elevate mitochondrial ROS production and PA additionally stimulates lipid peroxidation. These findings suggest that Prdx proteins may act to buffer treatment-induced oxidative stress and limit cytotoxic consequences. This is supported by our previous results showing significant induction of Prdx genes in response to oleuropein and punicic acid [29–31]. Similar effects have been reported in other cancer models, where suppression of Prdx family members enhanced intracellular ROS accumulation and significantly increased sensitivity to ROS-inducing chemotherapeutic agents and radiation, ultimately promoting apoptotic cell death [42–45]. Collectively, these studies support the notion that Prdx proteins are key determinants of resistance to oxidative stress and that their loss lowers the threshold for ROS-mediated cytotoxicity, providing a possible mechanistic explanation for the enhanced susceptibility of Prdx-deficient MCF-7 cells to both OL and PA observed in the present study.

Among the individual family members examined, Prdx3 appears particularly important. Prdx3 is localized primarily within mitochondria, where it detoxifies mitochondrial ROS [23]. Previous work from our lab showed that Prdx3 suppression in MCF-7 cells significantly increased susceptibility to doxorubicin, revealing its importance in counteracting drug-induced ROS [46]. In addition, others have shown that Prdx3 silencing induced anticancer properties in two triple negative breast cancer lines [47]. In fact, Prdx3 has already been proposed as a potential drug target in breast cancer [48]. Our observation that Prdx3 knockdown significantly increased susceptibility to both OL and PA is consistent with its established role in detoxifying mitochondrial ROS. Because mitochondrial oxidative stress appears to be a major contributor to the mechanism of action of both compounds, loss of mitochondrial antioxidant protection would be expected to further increase ROS accumulation and cellular damage. Similar protective effects observed following suppression of Prdx4 and Prdx5 suggest that multiple antioxidant pathways contribute to maintaining redox balance during treatment.

Several limitations should be considered when interpreting these findings. First, our conclusions are based on the particular concentrations and time points used in this study, which were consistent with other studies but limited in scope. Experiments using more concentrations and additional timepoints would provide for a more comprehensive analysis of the concentration response and kinetics of these compounds. Interestingly, a recent study examining oleuropein cytotoxicity in MCF7 cells shows much higher sensitivity to oleuropein, with IC50s below 50uM [49], although an earlier highly cited study shows concentration susceptibility that is more consistent with our findings [9]. These differences could be due to source, purity and stability of the compounds used, passage number of MCF-7 cells, culture conditions or other factors. Another limitation of our study is that oxidative stress was evaluated through mitochondrial ROS production and lipid peroxidation, while additional markers of oxidative damage and antioxidant activity were not examined. Although the results suggest oxidative stress as a mechanism underlying OL and PA induced cytotoxicity, further experiments using N-acetylcysteine (NAC) would be needed to confirm confirm that ROS is mediating the cytotoxic effects of oleuropein and punicic acid, or that it is responsible for the effects of Prdx suppression. An examination of key oxidative stress and apoptosis related proteins would help to elucidate the role of oxidative stress in these cellular effects. Finally, the effect of Prdx suppression on susceptibility to oleuropein and punicic acid was only measured by MTT assay. Further examination of the effect on cytotoxicity, mitochondrial superoxide levels and lipid peroxidation would provide more insight into the role of peroxiredoxins in this process.

While we demonstrate these effects in MCF-7 cells, parallel experiments were not performed in another breast cancer line in this study. However, others have demonstrated cytotoxicity in triple negative breast cancer lines [9, 48]. A recent study showed similar concentrations induced cytotoxicity in MDA-MB-231 and MDA-MB-468, with higher sensitivity in the MDA-MB-468 cells [50]. In addition to comparison between breast cancer lines, an investigation of the effects of these chemicals on a non-cancerous breast epithelial line like the MCF10A cell line would provide further insight into the cancer-specific effects of these compounds. Our previous studies did demonstrate that MCF-10A cells exhibit lower susceptibility to oleuropein than MCF-7 cells [29], but much higher susceptibility to punicic acid than MCF-7 cells [30]. The mechanism underlying this distinction will be extremely important to understand as we consider possible therapeutic potential of these compounds.

Overall, the findings demonstrate that oleuropein and punicic acid induce cytotoxic effects in MCF-7 breast cancer cells that are associated with increased mitochondrial ROS production and, for punicic acid, lipid peroxidation. Furthermore, suppression of multiple Prdx family members enhanced cellular sensitivity to these compounds, supporting a protective role for peroxiredoxin antioxidants in breast cancer cells. These results suggest that disruption of antioxidant defense mechanisms may potentiate the anti-cancer activity of OL and PA and highlight the importance of redox regulation in determining cellular responses to oxidative stress inducing therapies.

## Conclusion

This study demonstrates that oleuropein and punicic acid have cytotoxic effects in MCF-7 breast cancer cells and provides evidence that oxidative stress may be a key mechanism underlying these responses. Our findings indicate that both oleuropein and punicic acid disrupt cellular redox balance through increased mitochondrial superoxide, and punicic acid additionally contributes to lipid peroxidation in these cells. Our results further suggest that the peroxiredoxin antioxidant system may help to protect MCF-7 cells from treatment-induced cytotoxicity, as the suppression of multiple Prdx family members increased cellular susceptibility to both chemicals. Together, these findings suggest that peroxiredoxins may play an important role in maintaining redox homeostasis and limiting oxidative damage in breast cancer cells. Understanding the interaction between oxidative stress inducing compounds and cellular antioxidant defenses may provide valuable insight into potential therapeutic strategies that enhance the sensitivity of breast cancer cells to treatment. Future studies should further investigate the specific mechanisms by which individual peroxiredoxin family members regulate oxidative stress responses and influence susceptibility to anticancer agents.

## Supplementary Information

Below is the link to the electronic supplementary material.

Supplementary file1 Fig. S1 Morphology in Untreated or Control Treated MCF-7 Cells. MCF-7 cells were seeded at 2,000 cells per well and treated the following day with nothing (left) or 0.35% ethanol representing vehicle control (right). After 72 h, cells were imaged at 200X magnification using phase contrast microscopy

Supplementary file2 Fig. S2 Phase Contrast Microscopy corresponding to MitoSOX Images from Fig. 3. Representative phase contrast images corresponding to the fluorescence microscopy images of Fig. 3, of MCF-7 cells treated with control, 50 or 200 mg/mL Oleuropein (OL), or 2, 10, or 50 mg/mL Punicic Acid (PA) for 24 h. Following treatment, cells were stained with MitoSOX Red to detect mitochondrial superoxide production (red) and Hoescht nuclear stain to visualize nuclei (blue). Images were acquired at 200X magnification with phase contrast microscopy

Supplementary file3 Fig. S3 Phase Contrast Microscopy corresponding to the MTT Data of Fig. 6. MCF-7 cells were transfected with negative control siRNA or siRNA targeting individual Prdxs (Prdx1–Prdx6). 48 h after transfection, cells were treated with either 50 mg/mL Oleuropein (top) or 10 mg/mL Punicic Acid for 72 h. Wells were imaged at 40X with phase contrast microscopy before being used for the MTT assay

Supplementary file4

## Data Availability

All summarized data generated and analyzed during this study are included in this published article.
